# The *Leishmania donovani* Lipophosphoglycan Excludes the Vesicular Proton-ATPase from Phagosomes by Impairing the Recruitment of Synaptotagmin V

**DOI:** 10.1371/journal.ppat.1000628

**Published:** 2009-10-16

**Authors:** Adrien F. Vinet, Mitsunori Fukuda, Salvatore J. Turco, Albert Descoteaux

**Affiliations:** 1 INRS-Institut Armand-Frappier and Centre for Host-Parasite Interactions, Laval, Québec, Canada; 2 Department of Developmental Biology and Neurosciences, Tohoku University, Sendai, Miyagi, Japan; 3 Department of Biochemistry, University of Kentucky, Lexington, Kentucky, United States of America; Seattle Biomedical Research Institute, United States of America

## Abstract

We recently showed that the exocytosis regulator Synaptotagmin (Syt) V is recruited to the nascent phagosome and remains associated throughout the maturation process. In this study, we investigated the possibility that Syt V plays a role in regulating interactions between the phagosome and the endocytic organelles. Silencing of Syt V by RNA interference revealed that Syt V contributes to phagolysosome biogenesis by regulating the acquisition of cathepsin D and the vesicular proton-ATPase. In contrast, recruitment of cathepsin B, the early endosomal marker EEA1 and the lysosomal marker LAMP1 to phagosomes was normal in the absence of Syt V. As *Leishmania donovani* promastigotes inhibit phagosome maturation, we investigated their potential impact on the phagosomal association of Syt V. This inhibition of phagolysosome biogenesis is mediated by the virulence glycolipid lipophosphoglycan, a polymer of the repeating Galβ1,4Manα1-PO_4_ units attached to the promastigote surface via an unusual glycosylphosphatidylinositol anchor. Our results showed that insertion of lipophosphoglycan into ganglioside GM1-containing microdomains excluded or caused dissociation of Syt V from phagosome membranes. As a consequence, *L. donovani* promatigotes established infection in a phagosome from which the vesicular proton-ATPase was excluded and which failed to acidify. Collectively, these results reveal a novel function for Syt V in phagolysosome biogenesis and provide novel insight into the mechanism of vesicular proton-ATPase recruitment to maturing phagosomes. We also provide novel findings into the mechanism of *Leishmania* pathogenesis, whereby targeting of Syt V is part of the strategy used by *L. donovani* promastigotes to prevent phagosome acidification.

## Author Summary

Upon their internalization by macrophages, *Leishmania donovani* promastigotes inhibit phagolysosome biogenesis. This inhibition is mediated by the virulence glycolipid lipophosphoglycan (LPG), attached to the promastigote surface. We recently showed that the exocytosis regulator Synaptotagmin (Syt) V controls early steps of phagocytosis, and remains associated to the phagosome during the maturation process. Here, we show that Syt V contributes to phagolysosome biogenesis by regulating the acquisition of the hydrolase cathepsin D and the vesicular proton-ATPase. Insertion of LPG into lipid microdomains of the phagosome membrane excluded Syt V from phagosomes, enabling *L. donovani* promatigotes to inhibit the recruitment of the vesicular proton-ATPase to phagosomes, preventing their acidification. Collectively, our results provide novel insight into the mechanism of vesicular proton-ATPase recruitment to maturing phagosomes and reveal how the virulence glycolipid LPG contributes to the mechanism of *L. donovani* pathogenesis by preventing phagosome acidification.

## Introduction

Phagocytosis consists in the uptake and destruction of invading microorganisms, thereby playing an essential role in host defense against infection [1]. Following internalization, microbes end up in a vacuole, the phagosome, which engages in a maturation process involving highly regulated fusion and fission events with early and late endosomes, and with lysosomes [2],[3]. This leads to the acidification of the phagosome and the acquisition of an array of hydrolases, culminating in the generation of a highly microbicidal environment [4]. Soluble N-ethylmaleimide-sensitive factor protein attachment protein receptor (SNARE)-mediated membrane fusion events regulate phagosome maturation by facilitating interactions with the endocytic compartments [5]. Hence, VAMP3 and syntaxin 13 are present transiently on the young phagosome to regulate early maturation steps, whereas VAMP7 and syntaxin 7 remain associated with the phagosome to regulate interactions with late endosomes/lysosomes [6]–[8]. The lysosome-associated Synaptotagmin (Syt) VII, which controls membrane delivery to nascent phagosomes [9], is also involved in phagolysosome fusion [9],[10]. Other components and partners of these SNARE fusion machineries required during phagosome maturation remain to be identified.

Phagolysosome biogenesis is an important means of controling microbial growth. Yet, several pathogenic microorganisms have evolved mechanisms to subvert the phagosome maturation process, thus avoiding an encounter with the macrophage microbicidal machinery including exposition to reactive oxygen species and to acidification [4],[11],[12]. Protozoan parasites of the genus *Leishmania* cause a spectrum of diseases in humans, ranging from self-healing ulcers to potentially fatal visceral leishmaniasis, which affect millions of people worldwide. *Leishmania* is transmitted to mammals under its promastigote form during the bloodmeal of infected sand flies. Following phagocytosis by macrophages, promastigotes must avoid destruction to differentiate into amastigotes, the mammalian stage of the parasite that replicate inside acidic and hydrolase-rich parasitophorous vacuoles [13]–[15]. To avoid the microbicidal arsenal of macrophages, *L. donovani* and *L. major* promastigotes create an intracellular niche through the inhibition of phagolysosome biogenesis [16]–[19]. Genetic and biochemical approaches established that this inhibition is strictly dependent on the presence of lipophosphoglycan (LPG), an abundant surface glycolipid consisting of a polymer of Galβ1,4Manα1-PO_4_ units anchored into the promastigote membrane via an unusual glycosyl phosphatidylinositol [20],[21]. Hence, phagosomes harboring LPG-defective promastigotes quickly mature into functional phagolysosomes and coating of the Galβ1,4Manα1-PO_4_-defective mutant *lpg2*-KO with purified LPG conferred the capacity to inhibit phagosome-lysosome fusion [16],[17],[19],[22],[23]. LPG-mediated phagosome remodeling is characterized by a periphagosomal accumulation of F-actin [22],[23] and by the exclusion of cytosolic components of the NADPH oxidase from the phagosome membrane [24]. By creating an environment devoid of oxidants, *L. donovani* promastigotes evade a major microbicidal mechanism of macrophages and can initiate their differentiation into amastigotes. The ability of LPG to inhibit phagosome maturation is consistent with its role in the establishment of *L. donovani* and *L. major* promastigotes inside macrophages [24],[25].

A possible mechanism by which LPG exerts its action on phagosome maturation involves the transfer of LPG from the parasite surface to lipid microdomains present in the phagosome membrane, causing a disorganization of these structures and preventing their formation after phagocytosis [26]–[29]. Phagosomal lipid microdomains are essential for the recruitment/assembly of the NADPH oxidase and the vacuolar proton-ATPase and are involved in the regulation of phagosome-endosome fusions [27],[30],[31]. Disruption of lipid microdomains by microbial virulence factors is likely to facilitate the establishment of infection through an effect on phagolysosomal biogenesis, as described for the cyclic β-1,2-glucans of *Brucella abortus* and the lipoarabinomannan of *Mycobacterium tuberculosis* [32],[33]. How lipid microdomains regulate interactions between phagosomes and the endocytic system is unclear. The fact that proteins involved in membrane fusion such as SNAREs and Syts are located in lipid microdomains is consistent with these structures acting as fusion sites [34],[35].

Recently, we identified the exocytosis regulator Syt V [36]–[39] as a recycling endosome-associated protein that is recruited to the forming phagosome independently of the phagocytic receptor engaged [40]. Silencing of Syt V by RNAi revealed a role for this protein during phagocytosis, particularly under conditions of high membrane demand, possibly through the mobilization of recycling endosomes as a source of endomembrane. The association of Syt V with the phagosome throughout the maturation process raised the possibility that Syt V regulates interactions with the endocytic system [40]. Here, we provide evidence for a novel function of Syt V in phagolysosome biogenesis, where it controls the acquisition of cathepsin D and the vesicular proton-ATPase. We also provide novel insight into the mechanism of *L. donovani* pathogenesis with the demonstration that insertion of LPG into GM1-containing microdomains impairs the association of Syt V to phagosome membranes, enabling *L. donovani* promatigotes to inhibit the recruitment of the vesicular proton-ATPase to phagosomes, thereby preventing their acidification.

## Results

### Silencing of Syt V impairs phagosomal recruitment of the vacuolar ATPase and cathepsin D

**Figure 1:**
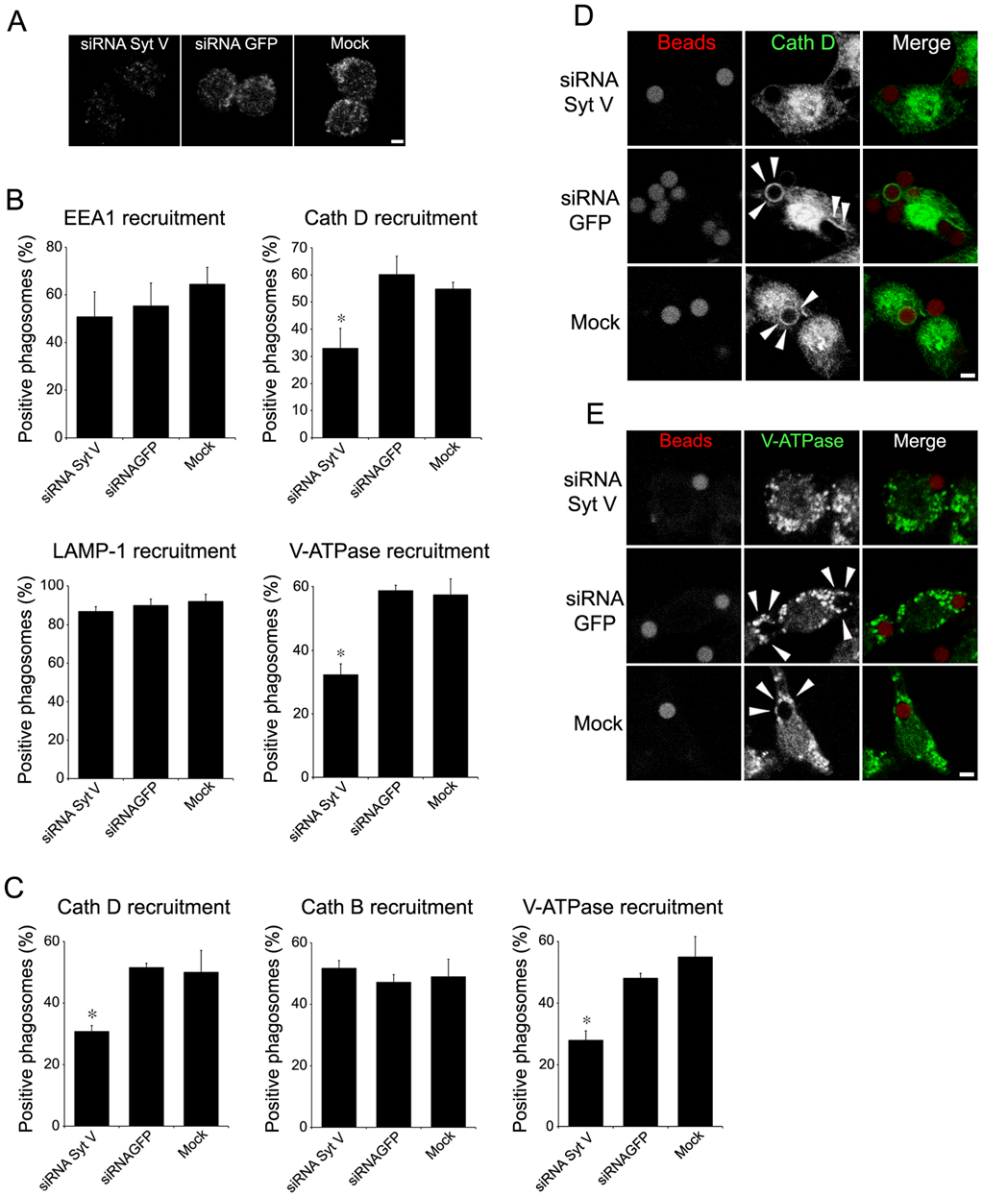
Silencing of Syt V impairs the recruitment of cathepsin D and the V-ATPase to phagosomes. RAW 264.7 cells were transfected with either a siRNA to Syt V, a siRNA to GFP, or only mock transfected, and incubated for 24 h. Efficiency of Syt V silencing was verified by confocal immunofluorescence microscopy (*A*). *B* and *C*, RAW 264.7 cells were allowed to internalize Zym (*B*) or latex beads (*C*) after siRNA transfection. Phagosomal recruitments were determined at 15 min for EEA1 and at 2 h for cathepsin D, cathepsin B, and LAMP1 on at least 100 phagosomes for each condition. Data are shown as the percentage of phagosomes showing recruitment. Five independent experiments were performed and the bars show the standard deviations of one representative triplicate (*, *p*≤0.05). *D* and *E*, representative confocal images illustrating the recruitment of cathepsin D (*D*) and of the V-ATPase (*E*) on phagosomes containing latex beads. Bar, 3 µm.

Syt V, a regulator of exocytosis, is recruited to the nascent phagosome and remains associated throughout the maturation process [40], suggesting that it may participate in the regulation of phagolysosome biogenesis. Maturing phagosomes sequentially interact with various endocytic organelles to acquire hydrolases such as cathepsins and the proton-vacuolar ATPase (V-ATPase), which is responsible for phagosome acidification [2],[41],[42]. To assess the potential role of Syt V in the acquisition of microbicidal features, we inhibited its expression by transfecting RAW 264.7 cells with a siRNA to Syt V [40] (Figure 1A) and we examined the localization of phagosomal markers following the internalization of Zymosan (Zym) or latex beads. Our results show that in the absence of Syt V, recruitment of both the early endosomal (EEA1) and the lysosomal (LAMP1) markers to Zym-containing phagosomes was normal (Figures 1B and S1A and B), whereas the acquisition of cathepsin D and the V-ATPase *c* subunit was inhibited (Figure 1B–E). Reduction in cathepsin D acquisition ranged from 25 to 35% for phagosomes containing beads and from 41 to 48% for phagosomes containing Zym, in five independent experiments. In the case of the V-ATPase *c* subunit, the reduction ranged from 30 to 50% for phagosomes containing beads and from 45 to 60% for phagosomes containing Zym in five independent experiments (Figure 1C–E). Interestingly, silencing of Syt V had no detectable effect on the acquisition of cathepsin B (Figure 1C). These results provide evidence that Syt V selectively regulates the phagosomal acquisition of cathepsin D and the V-ATPase *c* subunit.

### *L. donovani* promastigotes impair the phagosomal association of Syt V

**Figure 2:**
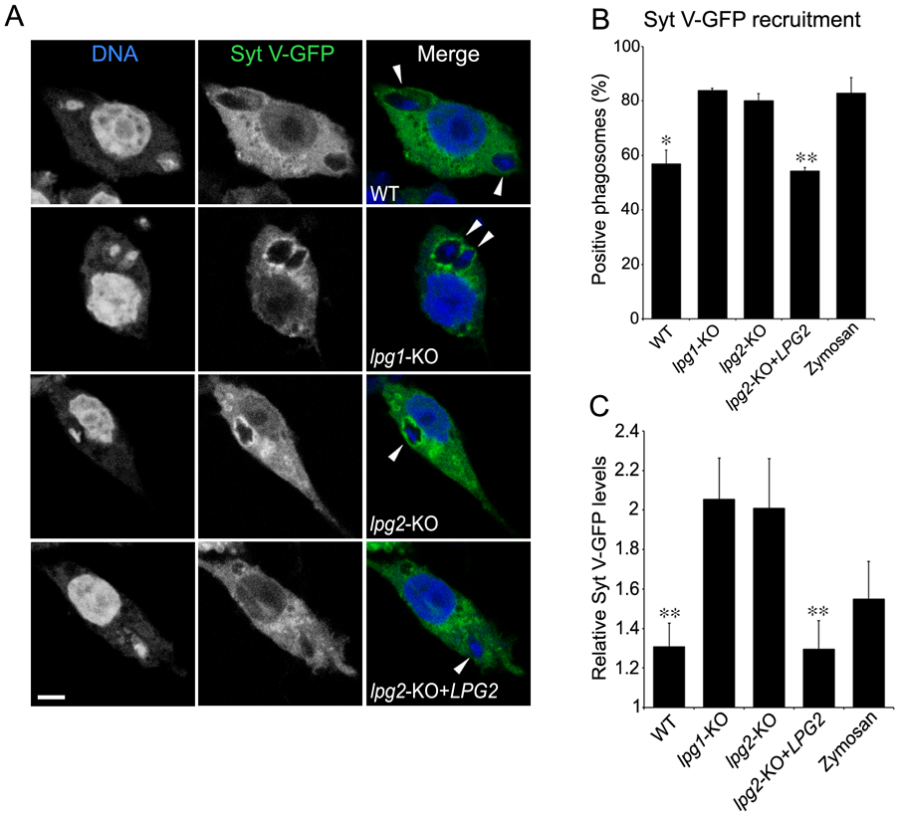
Syt V-GFP is excluded from *L. donovani* promastigote-containing phagosomes. Syt V-GFP RAW 264.7 cells were infected with either WT *lpg1*-KO, *lpg2*-KO, *lpg2*-KO+*LPG2 L. donovani* promastigotes or zymosan for 2 h, fixed and stained for DNA. *A*, Confocal images illustrating the presence of Syt V-GFP on parasite-containing phagosomes (shown by arrowheads). Presence (*B*) and relative levels (*C*) of Syt V-GFP were determined. Recruitment was determined on at least 100 phagosomes for each condition and expressed as a percentage of recruitment, and relative levels were determined by the P/C ratio as described in *Materials and Methods*. Three independent experiments were performed and the bars show the standard deviations of one representative triplicate (*, *p*≤0.05; **, *p*≤0.005; *B* and *C*, *p* values compare the presence and the accumulation of Syt V-GFP on phagosomes containing WT and *lpg2*-KO+*LPG2 vs lpg1*-KO and *lpg2*-KO parasites). Bar, 3 µm.

Given their ability to inhibit phagosome maturation [16],[17],[24], we explored the impact of *L. donovani* promastigotes and their LPG on the phagosomal association of Syt V. Accordingly, we infected the mouse macrophage cell line RAW 264.7 stably expressing a Syt V-GFP fusion protein (Syt V-GFP RAW 264.7 cells) with either wild-type (WT) *L. donovani* promastigotes, the LPG-defective *lpg1*-KO mutant, the Galβ1,4Manα1-PO_4_-defective *lpg2*-KO mutant or the *lpg2*-KO add-back (*lpg2*-KO+*LPG*2). We used Zym as a positive control for the recruitment of Syt V to phagosomes [40]. Our results show that Syt V-GFP was present on over 80% of phagosomes containing either *lpg1*-KO promastigotes, *lpg2*-KO promastigotes, or Zym (Figure 2A and B). In contrast, we detected Syt V-GFP on 54 to 65% of phagosomes containing either WT or *lpg2*-KO+*LPG*2 promastigotes in three independent experiments. Quantification analyses revealed a three-fold reduction in the levels of Syt V-GFP present on those positive phagosomes with respect to phagosomes containing either *lpg1*-KO or *lpg2*-KO promastigotes (Figure 2C). These observations suggested that LPG impairs the phagosomal recruitment of Syt V.

**Figure 3:**
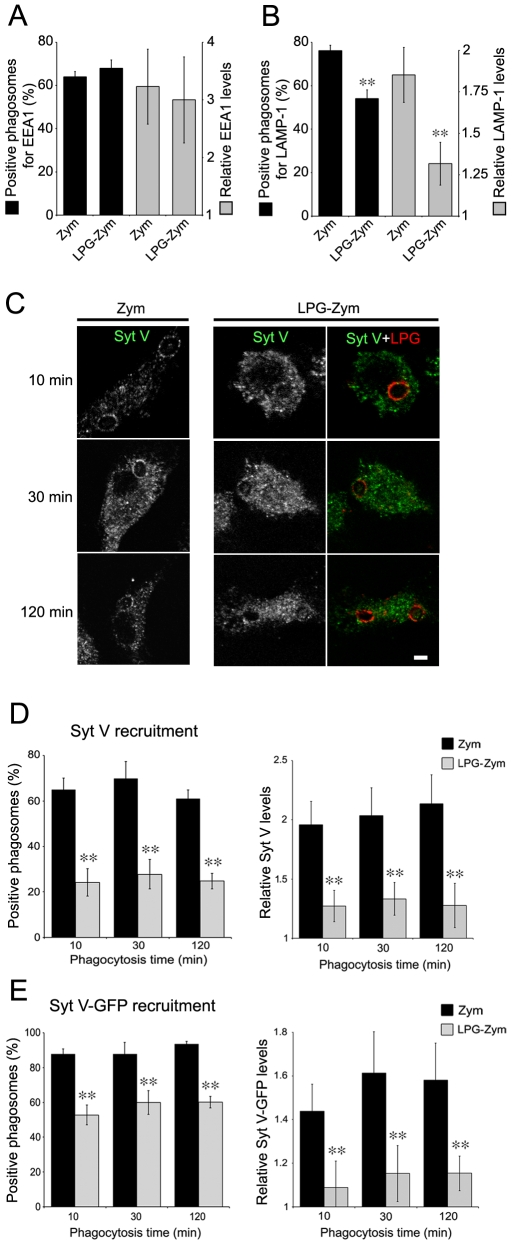
Recruitment of Syt V is impaired on phagosomes containing LPG-coated Zymosan. *A* and *B*, BMM were allowed to internalize Zym or LPG-Zym during 15 min (A) or 2 h (B), and prepared for confocal analysis. Presence (left y axis) and P/C ratio levels (right y axis) were determined for EEA1 (A) or LAMP-1 (B). *C* and *D*, BMM cells were allowed to internalize Zym or LPG-Zym for 10 min, 30 min or 2 h, fixed and stained for either endogenous Syt V (green) and LPG (red). The presence of Syt V and LPG on phagosomes is illustrated by confocal images (C). *D*. Quantification of Syt V recruitment (left panel) and relative Syt V levels (P/C ratio) on these phagosomes (right panel) were determined. *E*. Syt V-GFP cells were allowed to internalize Zym or LPG-Zym for 10 min, 30 min or 2 h, fixed and stained for LPG. Quantification of Syt V- recruitment (left panel) and relative Syt V levels on these phagosomes (right panel) were determined. The recruitment of EEA1, LAMP1 and Syt V was determined on at least 100 phagosomes for each condition, at least three independent experiments were performed and the bars show the standard deviations of one representative triplicate (*, *p*≤0.05; **, *p*≤0.005). Bar, 3 µm.

**Figure 4:**
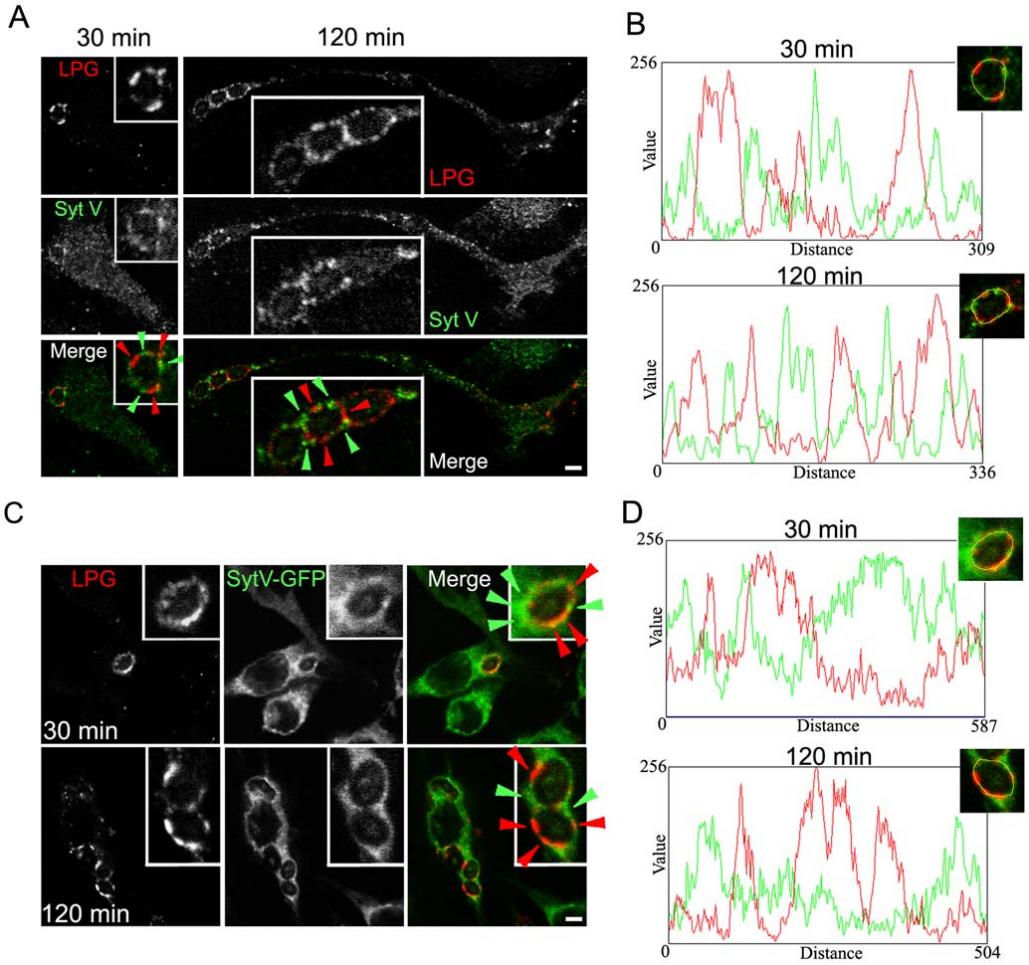
Exclusion of Syt V is restricted to the LPG insertion sites on the phagosome membrane. *A* and *B*, BMM were allowed to internalize Zym or Zym-LPG during 30 min or 2 h, and stained for Syt V (green) and LPG (red). Green arrowheads indicate a localized Syt V recruitment on phagosome membrane and red arrowheads indicate a localized LPG insertion into phagosome membrane (A). *C* and *D*, Syt V-GFP cells were allowed to internalize Zym or Zym-LPG for 30 min or 2 h, fixed and stained for LPG (red). Green arrowheads indicate a localized Syt V-GFP recruitment on phagosome membrane and red arrowheads indicate a localized LPG insertion into phagosome membrane (C). A rim around a representative phagosome formed in BMM (B) or in SytV-GFP cells (D) from *A* and *C* respectively, was manually traced with a one pixel width and fluorescence intensity profile of Syt V in green and LPG in red were represented in a graph for each phagocytosis time point. Bar, 3 µm.

To directly address the impact of LPG on the recruitment of Syt V to phagosomes, we fed bone marrow-derived macrophages (BMM) with either Zym or Zym coated with purified LPG (LPG-Zym) [22]. Consistent with previous observations [17],[22], we found a reduced acquisition of LAMP-1 on phagosomes containing LPG-Zym, whereas the recruitment of EEA1 to phagosomes containing Zym or LPG-Zym was similar (Figure 3A and B). In the case of Syt V, we detected its presence on 24 to 30% of phagosomes containing LPG-Zym compared to over 60% of phagosomes containing Zym at all time points tested in three independent experiments (Figure 3C and D). Quantification analyses showed that the levels of Syt V present on those positive phagosomes containing LPG-Zym was significantly lower than the Syt V levels on phagosomes containing Zym (Figure 3C and D). We obtained similar results with the Syt V-GFP RAW 264.7 cells (Figures 3E and S2). Furthermore, the signals for Syt V (green) and LPG (red) rarely superimposed on the phagosome membrane (Figure 4A), and fluorescence intensity line scans acquired along the periphery of phagosomes showed that the most intense LPG and Syt V signals never overlapped, at both 30 min and 120 min after the initiation of phagocytosis (Figure 4B). We made similar observations in Syt V-GFP RAW 264.7 cells (Figure 4C and D). Collectively, these results established that insertion of LPG into the phagosomal membrane caused the exclusion of Syt V in a very localized manner.

### Recruitment of Syt V to GM1-containing microdomains of phagosome membranes is prevented by LPG

**Figure 5:**
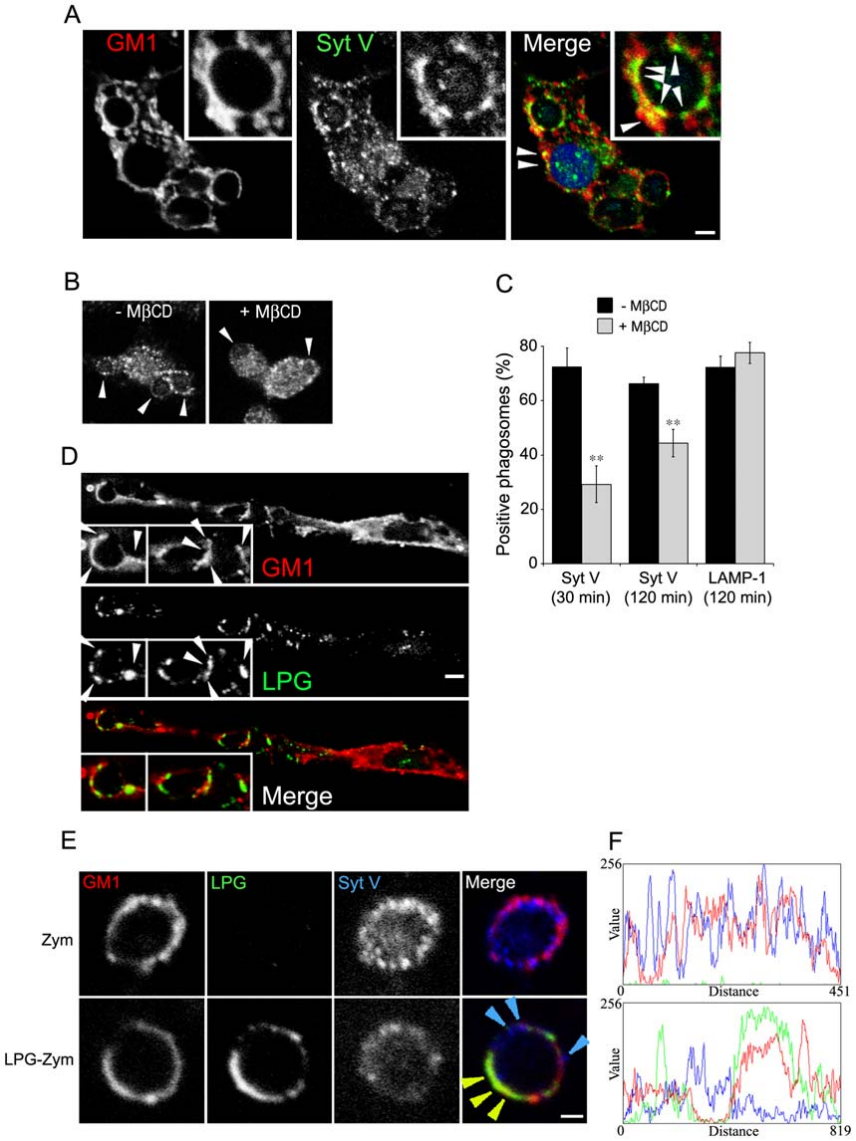
Recruitment of Syt V to GM1-containing microdomains of phagosome membranes is prevented by LPG. *A*, BMM were allowed to internalize Zym for 30 min, fixed and stained for endogenous Syt V (green) and GM1 (red). White arrowheads indicate examples of colocalization between Syt V and GM1-positive microdomains, indicating a Syt V enrichment on these microdomains. *B* and *C*, BMM were either left untreated or treated with 10 mmol/L MβCD for 1 h before the internalization of Zym for 30 and 120 min. Cells were then fixed and stained for Syt V and LAMP-1. Representative confocal images of Syt V recruitment on cells with or without MβCD treatment is presented (*B*), white arrowheads indicate phagosomes. Syt V acquisition is expressed as a percentage of phagosome recruitment for Syt V. At least 100 phagosomes for each condition were assessed. Three independent experiments were performed and the bars show the standard deviations of one representative triplicate (*C*) (**, *p*≤0.005). *D*, BMM were allowed to internalize Zym-LPG for 30 min, fixed and stained for LPG (green) and GM1 (red). White arrowheads indicate a colocalization between LPG and GM1-positive rafts. BMM were allowed to internalize Zym (*E*, upper panel) or LPG-Zym (*E*, lower panel) for 30 min, fixed and stained for Syt V (blue), LPG (green) and GM1 (red). Blue arrowheads indicate a local Syt V acquisition on phagosome membrane and yellow arrowheads indicate a local colocalization between GM1 microdomains and LPG. A rim around each phagosome was manually traced with a one pixel width and fluorescence intensity profile of Syt V in blue, LPG in green and GM1 in red were represented in a graph (*F*). Bars, 3 µm (*A*, *B* and *D*) or 1 µm (*E*).

In rat brain synaptosomes, a fraction of Syt I and Syt II is present in lipid rafts [34]. To examine whether LPG-mediated exclusion of Syt V from phagosomes was related to the insertion of LPG into lipid microdomains [27],[29] (Figure 5D), we first determined whether phagosome-associated Syt V was present in these microdomains. Our results clearly show that a fraction of Syt V colocalizes with GM1-microdomains on Zym-containing phagosomes (Figure 5A, arrowheads). Consistently, cholesterol depletion by methyl-β-cyclodextrin inhibited the recruitment of Syt V (Figure 5B and C). Having established that phagosomal Syt V associates with GM1-containing microdomains, we examined the localization of LPG, Syt V, and GM1 on phagosomes containing either Zym or LPG-Zym. For phagosomes containing Zym, the signals for Syt V (blue) and GM1 (red) superposed to a large extent and fluorescence intensity line scans acquired along the periphery of a representative phagosome showed that most of the Syt V and GM1 signals overlapped (Figure 5E and F, top panel). In contrast, on phagosomes containing LPG-Zym, the signals for LPG and GM1 colocalized, whereas most of the remaining Syt V signal was not associated with GM1 (representative phagosome, Figure 5E and F, bottom panel). These results established that association of LPG with GM1-containing microdomains resulted in the exclusion or dissociation of Syt V from the phagosome membrane.

### *L. donovani* promastigotes exclude the V-ATPase from phagosomes via their LPG

**Figure 6:**
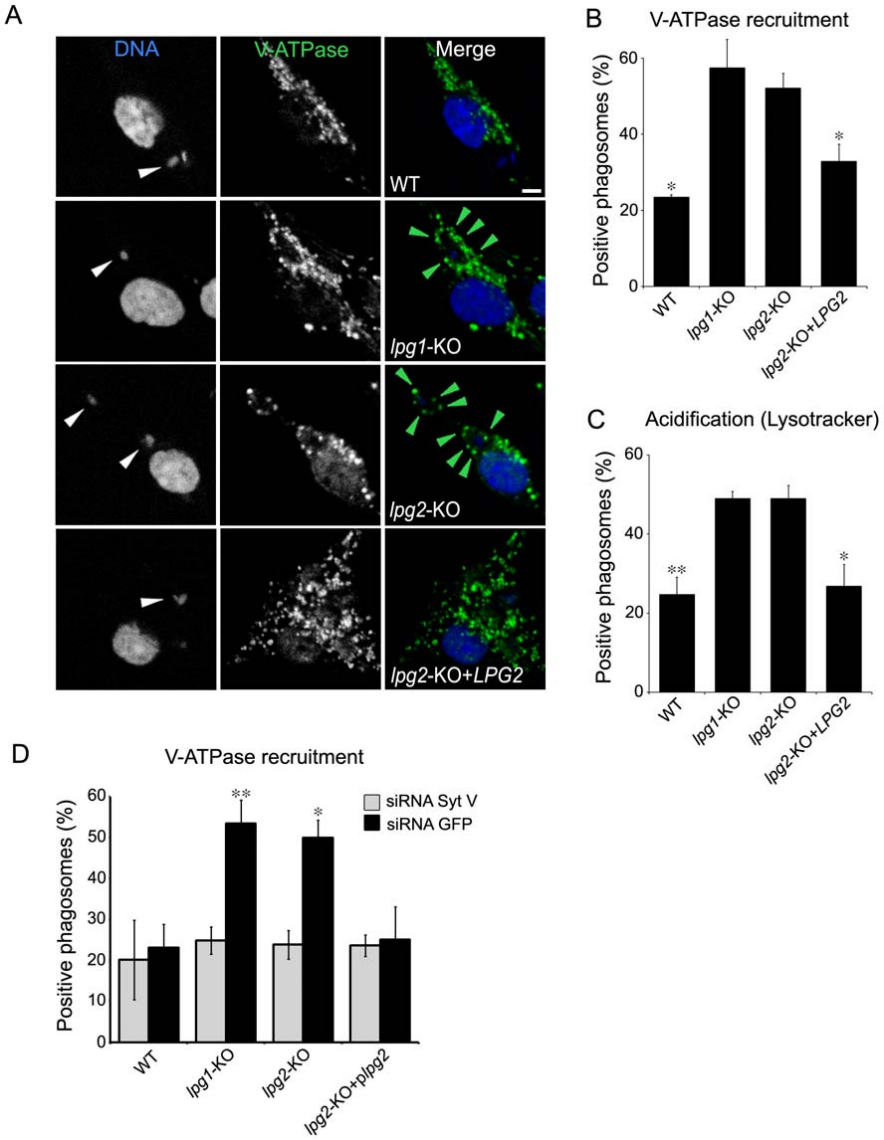
LPG prevents delivery to phagosomes of the V-ATPase and acidification through a Syt V-dependent mechanism. *A* and *B*, BMM cells were infected with either WT, *lpg1*-KO, *lpg2*-KO, *lpg2*-KO+*LPG2* promastigotes for 2 h, fixed and stained for V-ATPase (green) and DNA (blue). *A*, Confocal images illustrating V-ATPase acquisition (green arrowheads) on parasite-containing phagosomes (white arrowheads). *B*, V-ATPase acquisition was determined on at least 100 phagosomes for each condition. Three independent experiments were performed and the bars show the standard deviations of one representative triplicate (*, *p*≤0.05; *p* values compare the presence and the relative levels of V-ATPase on phagosomes containing WT and *lpg2*-KO+*LPG2 vs lpg1*-KO and *lpg2*-KO parasites). *C*, BMM cells were incubated 2 h with Lysotracker red prior to infection with either WT, *lpg1*-KO, *lpg2*-KO, *lpg2*-KO+*LPG2* promastigotes for 2 h and then fixed. *D*, RAW 264.7 cells were transfected with either a siRNA to Syt V or a siRNA to GFP, incubated for 24 h and infected with either WT, *lpg1*-KO, *lpg2*-KO, *lpg2*-KO+*LPG2* promastigotes for 2 h. Macrophages were then fixed and stained for DNA and the V-ATPase. Phagosomal recruitments were determined on at least 60 phagosomes for each condition. Two independent experiments were performed and the bars show the standard deviations of one representative triplicate. Data are shown as the percentage of recruitment (*, *p*≤0.05; **, *p*≤0.005; *p* values compare the acquisition of V-ATPase on phagosomes containing WT and *lpg2*-KO+*LPG2 vs lpg1*-KO and *lpg2*-KO parasites). Bar, 3 µm.

**Figure 7:**
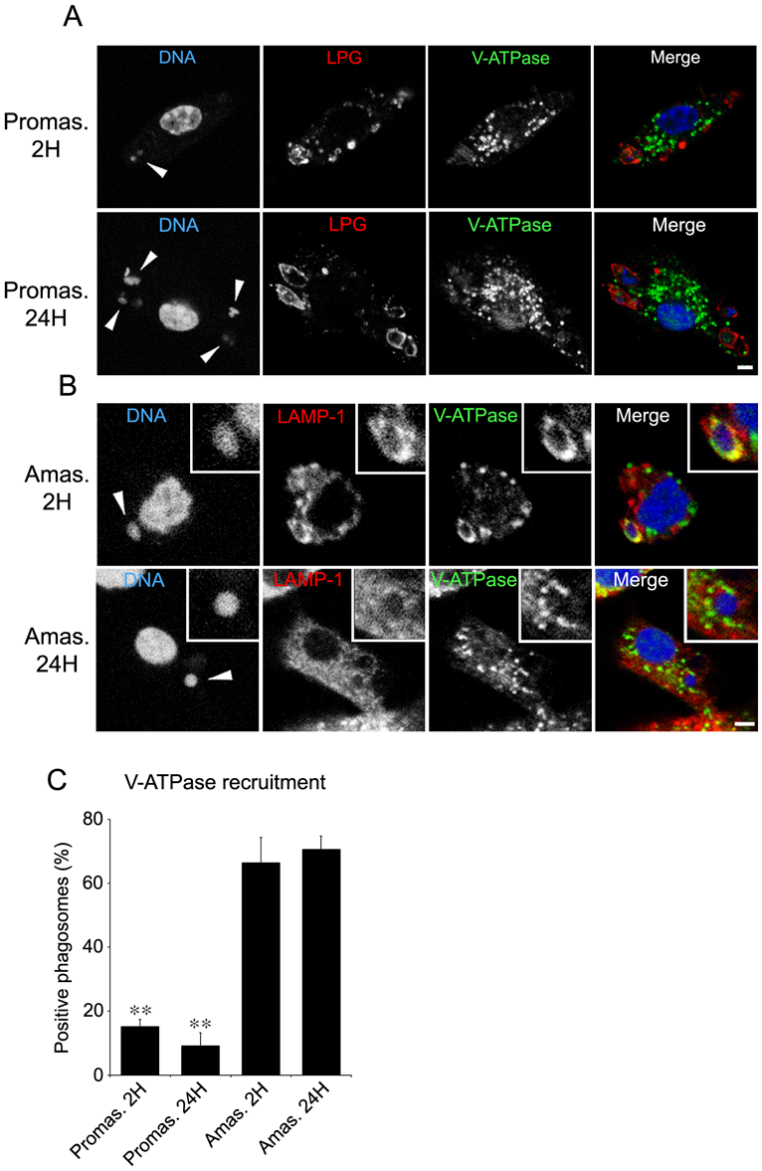
The inhibition of the V-ATPase acquisition on phagosomes is specific for the promastigote stage. *A*–*C*, BMM cells were infected with either WT promastigotes or amastigotes for 2 h and 24 h, fixed and stained for V-ATPase (green), LPG (red) and DNA (blue) (A) or V-ATPase (green), LAMP-1 (red) and DNA (blue) (B). *A* and *B*, Confocal images illustrating V-ATPase acquisition on parasite-containing phagosomes (white arrowheads). *C*, V-ATPase acquisition was determined on at least 100 phagosomes for each condition and expressed as a percentage of recruitment. Three independent experiments were performed and the bars show the standard deviations of one representative triplicate (**, *p*≤0.005; *p* values compare the acquisition of V-ATPase on phagosomes containing promastigotes *vs* amastigotes parasites). Bar, 3 µm.

The demonstration that Syt V regulates acquisition of the V-ATPase led us to verify the hypothesis that exclusion or dissociation of Syt V from phagosomes containing *L. donovani* promastigotes may impair the recruitment of the V-ATPase to these phagosomes. At 2 h after the initiation of phagocytosis, our results from three independent experiments showed a reduction in the recruitment of the V-ATPase *c* subunit on phagosomes containing WT promastigotes, ranging from 54 to 62% with respect to phagosomes containing either *lpg1*-KO or *lpg2*-KO promastigotes (Figure 6A and B). Co-localization of the V-ATPase *c* subunit with LAMP-1 on phagosomes containing *lpg1*-KO promastigotes showed that the V-ATPase *c* subunit was present on the phagosome membrane (Figure S3). As expected, phagosomes containing *lpg2*-KO+*LPG*2 cells were similar to WT-phagosomes with respect to the presence of the V-ATPase. We next monitored the acidification of *L. donovani* promastigote-containing phagosomes using the lysosomotropic agent LysoTracker red as an indicator of phagosome pH. Our results showed a clear correlation between the presence of the V-ATPase *c* subunit and the association of LysoTracker red to phagosomes (Figure 6C). In Figure 1, we showed that silencing of Syt V inhibited recruitment of the V-ATPase *c* subunit to phagosomes containing Zym or latex beads. In Figure 6D, we show that silencing of Syt V abrogated recruitment of the V-ATPase *c* subunit to phagosomes containing *lpg1*-KO and *lpg2*-KO mutants. In the case of phagosomes containing either WT or *lpg2*-KO+*LPG*2 promastigotes, Syt V silencing had the same effect as the presence of LPG on the recruitment of the V-ATPase *c* subunit (Figure 6D). Collectively, these results show that LPG enables *L. donovani* promatigotes to inhibit phagosomal recruitment of the V-ATPase by a Syt V-dependent mechanism and to prevent acidification. Remarkably, at 24 h after the initiation of phagocytosis, we detected the V-ATPase *c* subunit on only 10 to 17% of phagosomes containing *L. donovani* promastigotes in three independent experiments, consistent with LPG still being present (Figure 7A and C). At this time point, we detected LysoTracker red on only 20% of phagosomes containing WT promastigotes (not shown), indicating that promastigotes remodel their intracellular niche to establish infection in a compartment that fails to acidify, at a time when differentiation into amastigotes takes place. In contrast, we detected the V-ATPase *c* subunit on 66 to 71% of phagosomes containing *L. donovani* amastigotes at both 2 h and 24 h after the initiation of phagocytosis (Figure 7B and C). This observation is consistent with the fact that amastigotes replicate in an acidic phagolysosomal compartment [14].

## Discussion

The exocytosis regulator Syt V is recruited to the nascent phagosome and remains associated throughout the maturation process [40], leading us to investigate its potential role in modulating interactions between the phagosome and endocytic organelles. Our results revealed that whereas silencing of Syt V had no effect on the recruitment of EEA1, LAMP-1, and cathepsin B, it inhibited the phagosomal acquisition of cathepsin D and of the V-ATPase *c* subunit. These findings indicated that Syt V plays a role in phagolysosome biogenesis, possibly by regulating the interaction between phagosomes and a subset of late endosomes or lysosomes enriched in cathepsin D and in the V-ATPase *c* subunit. Alternatively, Syt V may be needed to reach the level of phagosome maturation necessary to acquire the machinery that regulates the recruitment of cathepsin D and the V-ATPase *c* subunit. Our finding that acquisition of cathepsin B and cathepsin D is mediated by distinct mechanisms supports the demonstration that various hydrolases appear sequentially, at various time points during phagosome maturation [42]. This view is also consistent with evidence that various sub-populations of early endosomes, late endosomes, and lysosomes co-exist and that these compartments contain significant heterogeneity [43]. Together with previous findings [27], our results show that phagosomal acquisition of the V-ATPase and LAMP-1 are mediated through distinct mechanisms. Hence, the observations that LAMP-1 is recruited to phagosomes independently of Syt V and that *L. donovani* promastigotes (and LPG) impair the recruitment of LAMP-1 point to the existence of other inhibitory mechanisms and illustrate the complexity of phagolysosome biogenesis. The role of Syt V in regulating interactions between the phagosome and the endosomal compartments thus seems specific and further studies will be necessary to understand its precise role during phagosome maturation. Recent studies by Andrews and colleagues revealed that the lysosome-associated Syt VII, which controls membrane delivery to nascent phagosomes [9], is involved in phagolysosome fusion [9],[10]. It will be of interest to determine whether Syt V and Syt VII use similar mechanisms to regulate phagolysosome biogenesis.

To establish infection inside macrophages, *L. donovani* promastigotes, the form of the parasite transmitted to mammals by the sand fly vector, create an intracellular niche by inhibiting phagolysosome biogenesis [16]. Genetic and biochemical approaches revealed that this inhibition is mediated by the parasite surface glycolipid LPG [16],[17],[22]. Insight into the mechanism of this inhibition came from the observations that LPG transfers from the parasite surface to the nascent phagosome membrane [26], where it disrupts existing lipid microdomains and alters the formation of these structures after promastigote internalization [28],[29]. Whereas the precise mechanism remains to be elucidated, the current model is that LPG inserts into lipid microdomains via its GPI anchor, thereby allowing the negatively charged Galβ1,4Man-PO_4_ polymer of LPG to directly interfere with the clusterization of molecules into these microdomains. This model is consistent with the demonstration that alteration of membrane properties is dependent on the length of the Galβ1,4Man-PO_4_ polymer [16],[44]. Because of their role in clustering specific sets of proteins, membrane lipid microdomains are central to a wide variety of cellular processes, including regulated exocytosis [45],[46]. Our findings that Syt V was present in GM1-enriched phagosome microdomains and that LPG inserts into or associates with these structures to interfere with the phagosomal association of Syt V thus provides new insight into the mechanism of LPG-mediated inhibition of phagolysosome biogenesis.

Acquisition of an array of hydrolases and acidification of the phagosome enable the generation of a highly microbicidal environment [4] and the creation of a compartment competent for antigen processing and presentation [47]. To circumvent killing following uptake by macrophages, several intracellular microorganisms interfere with phagosome acidification and maturation [4],[12],[48]. The discovery that *L. donovani* promastigotes establish infection inside a compartment from which the V-ATPase is excluded may thus be favorable for parasite survival. Incidentally, a recent study showed that phagosome acidification is defective in Stat1^−/−^ macrophages and this correlated with an increased survival of *L. major* promastigotes, suggesting a role for acidic pH in the control of intracellular *Leishmania* growth early during infection [49]. Furthermore, the finding that phagosomes containing *L. donovani* promastigotes fail to acquire the V-ATPase and acidify even at 24 hours post-infection provides new insight on our undestanding of *Leishmania* biology. Indeed, in the absence of data on the pH of promastigote-containing phagosomes, it has been assumed that promastigotes initiate infection in an acidic environment and that differentiation of promastigotes into amastigotes is mainly triggered by a rapid exposure to an acidic environment and elevated temperature [50]. Exclusion of the V-ATPase raises the possibility that *L. donovani* promastigotes initiate the differentiation process in a non-acidified environment. Further studies will be required to fully address this point. An issue that remains unsolved pertains to the acquisition of phagolysosomal features and acidification of parasite-containing vacuoles upon completion of the differentiation of promastigotes into amastigotes. Indeed, previous work by Antoine and colleagues [14] established that *L. amazonensis* amastigotes reside within an acidic vacuole (pH 4.7–5.2), in agreement with the notion that *Leishmania* amastigotes are internalized within a vacuole that rapidly acquires lysosomal features and in which amastigotes proliferate [13],[51]. Consistent with these previous reports, we showed the presence of LAMP-1 and the V-ATPase *c* subunit on phagosomes containing *L. donovani* amastigotes as early as 2 h after internalization. A possible explanation is that during the first few days post-infection, the presence of LPG in the phagosome membrane prevents acidification and maturation, allowing promastigote-to-amastigote differentiation to take place. The down-regulation of LPG biosynthesis below detectable levels in amastigotes [52] may enable phagosomes to gradually acquire lysosomal features and to acidify.

Little is known on the mechanisms that regulate recruitment of the V-ATPase to maturing phagosomes. The identification of Syt V as a regulator of this process and the fact that Syt V is present in microdomains of the phagosome membrane is consistent with the notion that these structures are important for the recruitment of the V-ATPase to the phagosome membrane [27]. Of interest, the V-ATPase *c* subunit has been previously identified in Triton X-100-resistant fractions from rat brain synaptic vesicles in association with synaptobrevin 2 and synaptophysin [53], leading the authors of that study to conclude that this interaction may play a role in recruiting the V-ATPase to synaptic vesicles. Whether Syt V is part of such a SNARE complex on phagosomes and the characterization of this complex are important issues that await further investigation.

**Figure 8:**
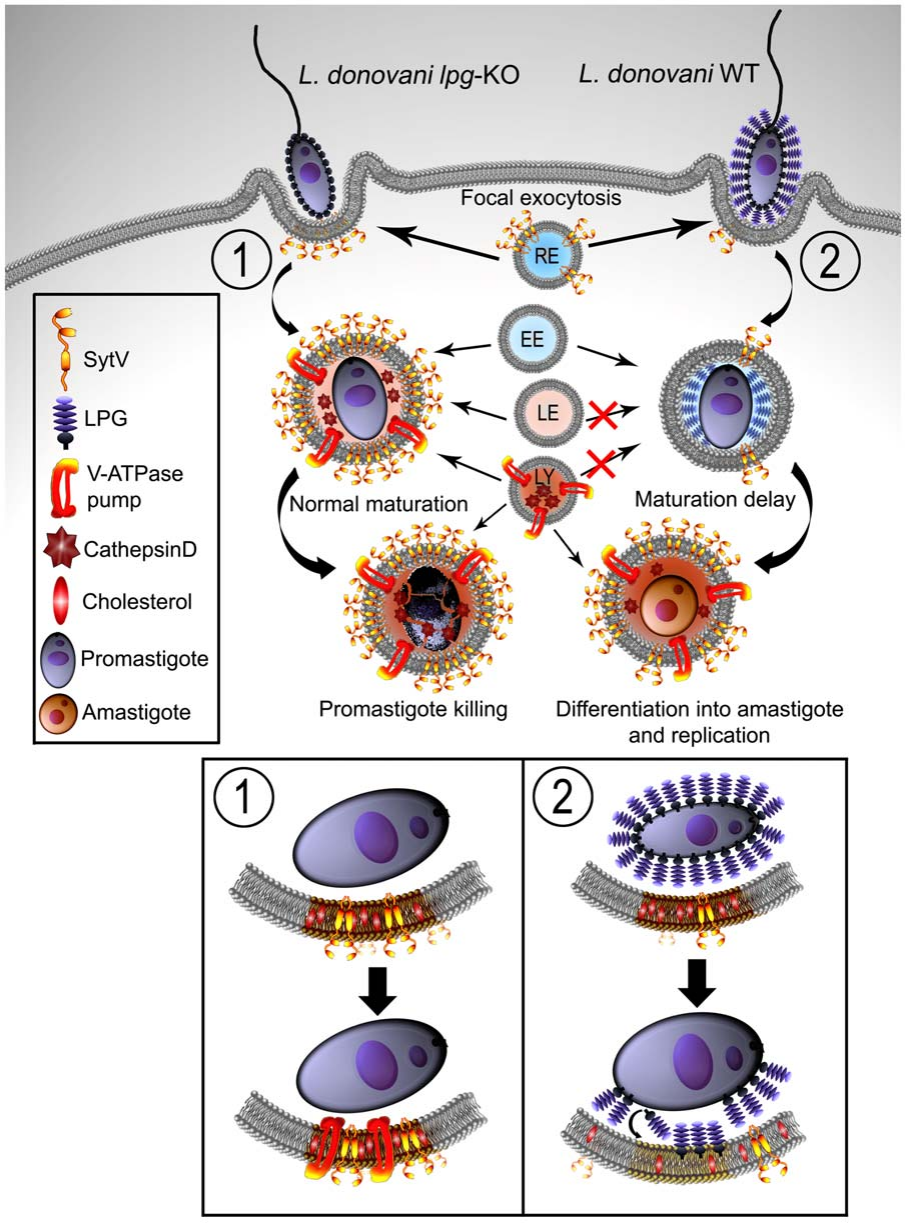
Working model for the exclusion of the V-ATPase from *L. donovani* promastigote-harboring phagosomes. Both WT and LPG-defective *L. donovani* promastigotes bind to macrophages, inducing the phagocytic process. During this step, membrane from internal vesicles such as recycling endosomes (RE) bearing Syt V participate to the membrane supply necessary for the formation of the nascent phagosome. LPG transfers to the phagosome membrane and inserts into lipid microdomains, excluding Syt V from the newly formed phagosome. Exclusion of Syt V impairs phagolysosome biogenesis, including acquisition of cathepsin D and V-ATPase (2) when compared to phagosomes harboring *L. donovani* promastigotes lacking LPG (1), which interact with the endosomal compartment (early endosomes, EE; late endosomes, LE; lysosomes, LY). This creates an intracellular niche that fails to acidify, at a time when promastigotes differentiate into amastigotes, which are resistant to the microbicidal properties of the phagolysosome. Mutants lacking LPG are degraded by the phagolysosomal environment.

In this study, we provided novel findings into the mechanism of *Leishmania* pathogenesis, whereby targeting of Syt V, which plays a role in the acquisition of phagosome microbicidal properties, is part of the strategy used by *L. donovani* promastigotes to create a niche propitious to the establishment of infection within mammalian hosts (see working model, Figure 8). Interestingly, phagocytosis of either zymosan or *lpg2*-KO promastigotes coated with the virulence glycolipid lipoarabinomannan from *Mycobacterium tuberculosis*, impaired the phagosomal association of Syt V (Figure S4). Whether other intracellular microorganisms use a similar mechanism to remodel their intracellular niche remains to be investigated.

## Materials and Methods

### Macrophages

All animals were handled in strict accordance with good animal practice as defined by the Canadian Council on Animal Care, and all animal work was approved by the Comité institutionel de protection des animaux of INRS- Institut Armand-Frappier (protocol 0811-08). BMM were obtained by growing bone marrow cells from female BALB/c mice at 37°C in 5% CO_2_ for 7 days in Dulbecco Modified Eagle Medium with L-glutamine (Life Technologies) supplemented with 10% heat-inactivated FBS (Hyclone, Logan, UT), 10 mM Hepes (pH 7.4) and antibiotics (complete medium) in the presence of 15% (v/v) L929 cell-conditioned medium as a source of colony-stimulating factor (CSF)-1 [54]. BMM were made quiescent by culturing them in the absence of CSF-1 for 18 h prior to being used. The murine macrophage cell line RAW 264.7 was grown in complete medium in a 37°C incubator with 5% CO_2_. Stably transfected RAW264.7 cells expressing Syt V-GFP (Syt V-GFP RAW 264.7 cells) were previously described [40]. Transfectants were cultured in complete medium containing 500 µg/ml G418 (Life Technologies).

### Parasites

*Leishmania donovani* promastigotes (Sudanese strain 1S) were grown at 26°C in RPMI 1640 medium supplemented with 20% heat-inactivated FBS, 100 µM adenine, 20 mM 2-[N-morpholino]ethanesulphonic acid (pH 5.5), 5 µM hemin, 3 µM biopterin, 1 µM biotin and antibiotics. The isogenic *L. donovani* LPG-defective mutants *lpg1*-KO and *lpg2*-KO were described previously [55]. The *lpg1*-KO mutant secretes repeating Galβ1,4Manα1-PO_4_-containing molecules, but lacks the ability to assemble a functional LPG glycan core [56], precluding synthesis of LPG. The *lpg2*-KO mutant expresses the truncated LPG Gal(α1,6)Gal>(α1,3)Gal*_f_*(β1,3)[Glc(α1-P)]Man(α1,3)Man(α1,4)GN(α1,6)-PI, and does not synthesize repeating Galβ1,4Manα1-PO_4_ units [57]. The *lpg2*-KO+*LPG2* add-back was grown in the presence of 50 µg/ml G418. For infections, promastigotes were used in late stationary phase of growth. *L. donovani* amastigotes (Strain LV9) were isolated from the spleen of infected female LVG Golden Syrian hamsters (Charles River, St-Constant, QC, Canada), as described [58].

### Reagents and antibodies

The rabbit anti-Syt V spacer antiserum was raised against the cytoplasmic region between the transmembrane and the C2 domain (aa 71–216) [37] and was affinity-purified. The rat monoclonal antibody against LAMP-1 developed by J. T. August (1D4B) was obtained through the Developmental Studies Hybridoma Bank at the University of Iowa, and the National Institute of Child Health and Human Development. The rabbit antiserum against the 16 kDa proteolipid subunit (*c* subunit) of the V_0_ sector of the V-ATPase was kindly provided by Dr. Mhairi Skinner (University of Guelph, ON, Canada) [59]. The mouse monoclonal antibody against EEA1 was from BD Transduction Laboratories. The rabbit antiserum against cathepsin B was from Millipore and the rabbit antiserum against cathepsin D was from Upstate. The mouse monoclonal anti-LPG (CA7AE) was prepared from hybridoma supernatant [60]. Methyl-β-cyclodextrin (MβCD) was from Sigma (St-Louis, MO, USA). LPG was isolated from the log phase cultures of *L. donovani* promastigotes as previously described [61],[62]. Purified lipoarabinomannan (LAM) from H37Rv strain of *Mycobacterium tuberculosis* was from Colorado State University (Fort Collins, CO, USA).

### RNA interference

Syt V silencing by RNAi was performed as previously described [40] using a small interfering RNA (siRNA) corresponding to nucleotides 94–112 of the Syt V cDNA [38], whereas a siRNA specific to GFP was used as a negative control [63]. Adherent RAW 264.7 cells were transfected with siRNA duplexes at a final concentration of 240 nM using OligoFectamine (Invitrogen) as described [63]. A BLAST search against the mouse genome sequence database was performed to ensure that the chosen siRNA sequences targeted only the mRNA of interest.

### Cholesterol depletion

Cholesterol depletion was achieved by incubating macrophages with 10 mmol/L methyl-β-cyclodextrin (MβCD) (Sigma) in serum-free medium at 37°C for 1 h. Cells were washed with PBS before particle internalization.

### Coating and opsonization of the particles

Purified LPG and LAM were sonicated and added to the particles at a final concentration of 25 µM in PBS, pH 7.3, incubated at 37°C for 1 h. Particles were washed and resuspended in complete medium prior to phagocytosis experiments. The efficiency of LPG coating was assessed by immunofluorescence using the anti-repeating unit antibody CA7AE. Complement opsonization of *L. donovani* promastigotes was done as described [23] and complement opsonisation of beads and zymosan was carried out by incubating the particles in DMEM supplemented with 10% mouse serum for 30 min at 37°C prior to phagocytosis.

### Phagocytosis assay

For synchronized phagocytosis assays, macrophages were incubated with particles at a particle-to-cell ratio of 15∶1 (unless otherwise specified) for 15 min at 4°C. Excess particles were removed by several thorough washes with DMEM and phagocytosis was triggered by transferring the cells to 37°C for the indicated time points before processing for microscopy.

### Immunofluorescence

Macrophages were fixed for 10 min in PBS containing 2% paraformaldehyde, permeabilized using 0.1% Triton X-100, and nonspecific binding to surface FcγR was blocked using 1% BSA, 2% goat serum, 6% milk, and 50% FBS. For immunostaining, cells were labeled with the appropriate combinations of primary antibodies or antisera (anti-Syt V, LAMP-1, EEA1, cathepsin D, cathepsin B, V-ATPase, LPG), and secondary antibodies (anti-rabbit, anti-mouse or anti-rat AlexaFluor 488, 568 or 647; Molecular Probes). DRAQ5 (Biostatus, Leicestershire, UK) was used to visualize macrophage and parasite nuclei and CTX-B-568 or 647 (Molecular Probes) was used to visualize GM1-enriched rafts. Syt V-GFP RAW 264.7 cells were fixed and directly incubated with DRAQ5 before being mounted or subjected to immunofluorescence. Of note, we used Syt V-GFP RAW 264.7 cells to localize Syt V following infection with *L. donovani* promastigotes because our antiserum against Syt V cross-reacts with *Leishmania* epitopes. All coverslips were mounted on glass slides with Fluoromount-G (Southern Biotechnology Associates). Detailed analysis of protein presence and localization on the phagosome was performed using an oil immersion Nikon Plan Apo 100 (N.A. 1.4) objective mounted on a Nikon Eclipse E800 microscope equipped with a Bio-Rad Radiance 2000 confocal imaging system (Bio-Rad, Zeiss). Images were obtained using appropriate filters, through the sequential scanning mode of the LaserSharp software (Bio-Rad Laboratories, Zeiss) with a Kalman filter of at least 6.

### Phagosome acidification

BMM were preloaded with the acidotropic dye LysoTracker Red (Molecular Probes, Eugene, OR) diluted in DMEM (1∶1000) for 2 h at 37°C. Cells were washed and infected with promastigotes for 2 h at 37°C as described in *Phagocytosis assay*. Cells were then rinsed, fixed with 2% paraformaldehyde for 10 min, washed and directly incubated 20 min with DRAQ5 before being mounted for confocal analysis.

### Quantification of phagocytosis and protein recruitment on phagosomes

To assess the recruitment of proteins of interest, we assessed the presence or absence of staining on the phagosome membrane for each protein, and at least 100 phagosomes were randomly scanned for each condition. To quantify the levels of Syt V and Syt V-GFP (Figures 2C, 3D and 3E), EEA1 (Figure 3A) or LAMP-1 (Figure 3B), we determined the relative staining intensity as follows. The 488 and 568 nm excitation channels (emission 515/30 and 600/40 respectively) were separated and the protein staining rim around each phagosome was manually traced with a one pixel width. The fluorescence intensity of individual pixels was determined using the software Image J and an average intensity was calculated for each fluorescence intensity profile. To normalize intensity values of all phagosomes, cytosol intensity was also evaluated in the proximity area of the phagosome under study but far enough from the phagosome membrane to avoid quantifying residual phagosome fluorescence. Final phagosome intensity was expressed as the ratio of phagosome intensity (P) on cytosol intensity (C), thus P/C. In all cases, we ensured that signal intensity was not at saturation and the 20 more intense staining for each condition were selected and the average compared for the intensity level of each protein.

### Statistical analyses

Statistical analyses were performed using Student's two-tail two-sample unequal variance test.

## Supporting Information

Figure S1 Kinetics of EEA1 and LAMP-1 phagosomal recruitment are normal in the absence of Syt V. *A.* Representative confocal images illustrating EEA1 recruitment at 10 min of phagocytosis and LAMP-1 recruitment at 120 min of phagocytosis. Bar, 3 µm. *B.* RAW 264.7 cells were transfected with siRNAs to either Syt V or GFP, and incubated for 24 h. Cells were allowed to internalize Zym and phagosomal recruitments were determined at 10, 30, 60 and 120 min for EEA1 and LAMP1 on at least 100 phagosomes for each condition. Two independent experiments were performed and the bars show the standard deviations of one representative triplicate.
(3.91 MB PDF)

Figure S2 Recruitment of Syt V-GFP is reduced on phagosomes containing LPG-coated zymosan. Syt V-GFP cells were allowed to internalize Zym or LPG-Zym for 10 min, 30 min or 2 h, fixed and stained for LPG (red). Recruitment and relative levels of Syt V-GFP on phagosomes are illustrated by confocal images. Bar, 3 µm.
(1.40 MB PDF)

Figure S3 V-ATPase recruitment on phagosome membrane containing *lpg*-deficient promastigotes. BMM cells were infected with *lpg1*-KO promastigotes for 2 h, fixed and stained for the V-ATPase c subunit (green), LAMP-1 (red) ,and DNA (blue). The V-ATPase *c* subunit is present on the phagosome membrane, which is also positive for LAMP-1.
(1.15 MB PDF)

Figure S4 Recruitment of Syt V is prevented on phagosomes containing particles coated with the *Mycobacterium tuberculosis* lipoarabinomannan. *A* and *B*, SytV-GFP cells were allowed to internalized Zym or LAM-Zym (A), *lpg2*-KO or LAM-*lpg2*-KO (B) for 30 min or 2 h. The presence (*A* and *B*, left graph) and relative levels (*A* and *B*, right graph) of Syt V-GFP were determined. Three independent experiments were performed and the bars show the standard deviations of one representative triplicate (*, *p*≤0.05; **, p≤0.005).
(0.66 MB PDF)
