# Supplementary figures and images for: The Leishmania donovani Lipophosphoglycan Excludes the Vesicular Proton-ATPase from Phagosomes by Impairing the Recruitment of Synaptotagmin V
Source: PLoS Pathog. 2009 Oct 16;5(10):e1000628. doi: 10.1371/journal.ppat.1000628 (PMC2757729; doi:10.1371/journal.ppat.1000628)

A

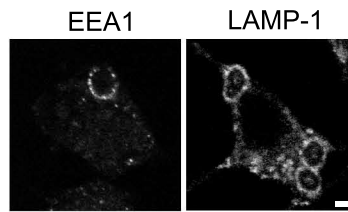

B

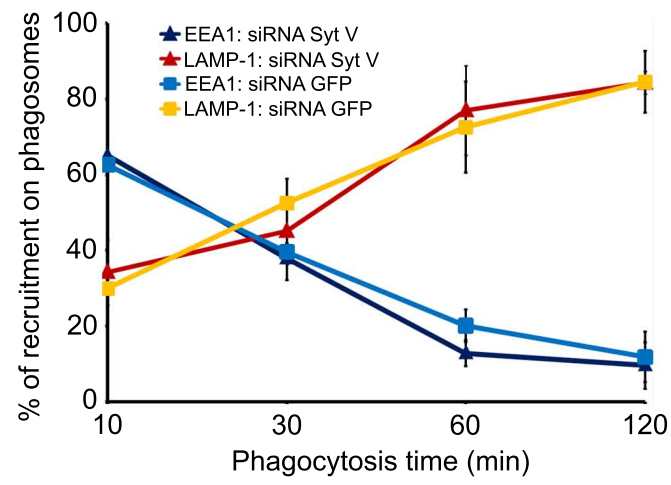

Supplement: Figure S1 — Kinetics of EEA1 and LAMP-1 phagosomal recruitment are normal in the absence of Syt V. A. Representative confocal images illustrating EEA1 recruitment at 10 min of phagocytosis and LAMP-1 recruitment at 120 min of phagocytosis. Bar, 3 µm. B. RAW 264.7 cells were transfected with siRNAs to either Syt V or GFP, and incubated for 24 h. Cells were allowed to internalize Zym and phagosomal recruitments were determined at 10, 30, 60 and 120 min for EEA1 and LAMP1 on at least 100 phagosomes for each condition. Two independent experiments were performed and the bars show the standard deviations of one representative triplicate. (3.91 MB PDF) [file ppat.1000628.s001.pdf]

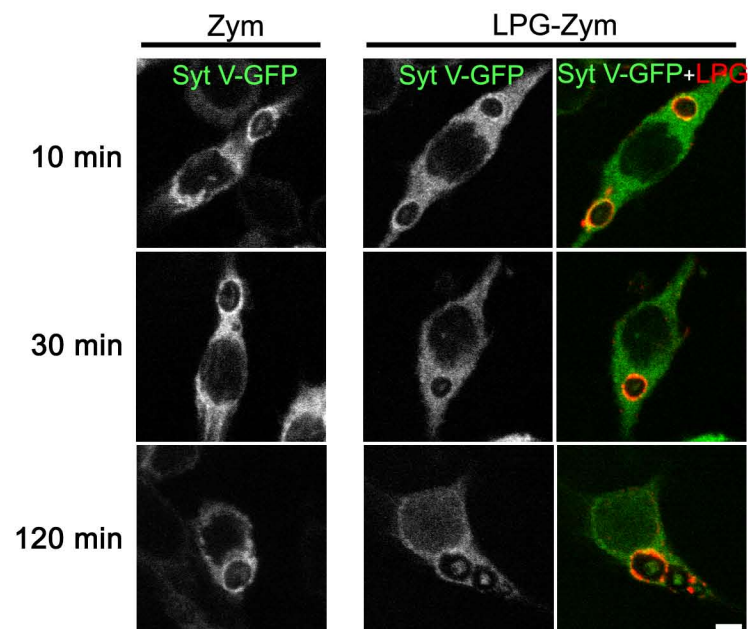

Supplement: Figure S2 — Recruitment of Syt V-GFP is reduced on phagosomes containing LPG-coated zymosan. Syt V-GFP cells were allowed to internalize Zym or LPG-Zym for 10 min, 30 min or 2 h, fixed and stained for LPG (red). Recruitment and relative levels of Syt V-GFP on phagosomes are illustrated by confocal images. Bar, 3 µm. (1.40 MB PDF) [file ppat.1000628.s002.pdf]

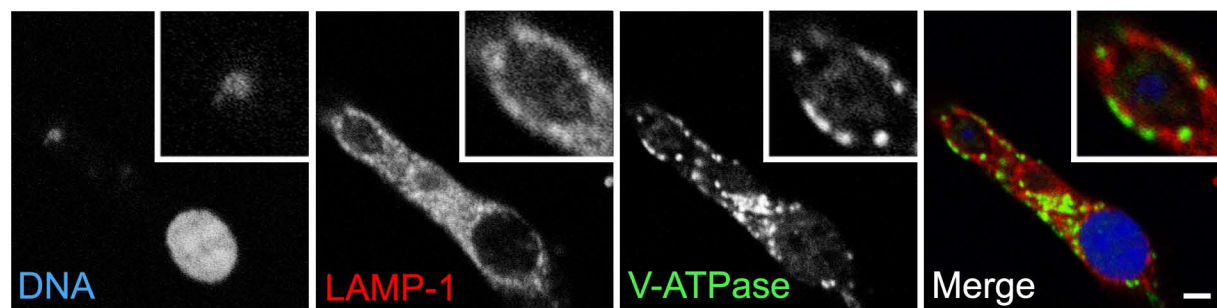

Supplement: Figure S3 — V-ATPase recruitment on phagosome membrane containing lpg-deficient promastigotes. BMM cells were infected with lpg1-KO promastigotes for 2 h, fixed and stained for the V-ATPase c subunit (green), LAMP-1 (red) ,and DNA (blue). The V-ATPase c subunit is present on the phagosome membrane, which is also positive for LAMP-1. (1.15 MB PDF) [file ppat.1000628.s003.pdf]

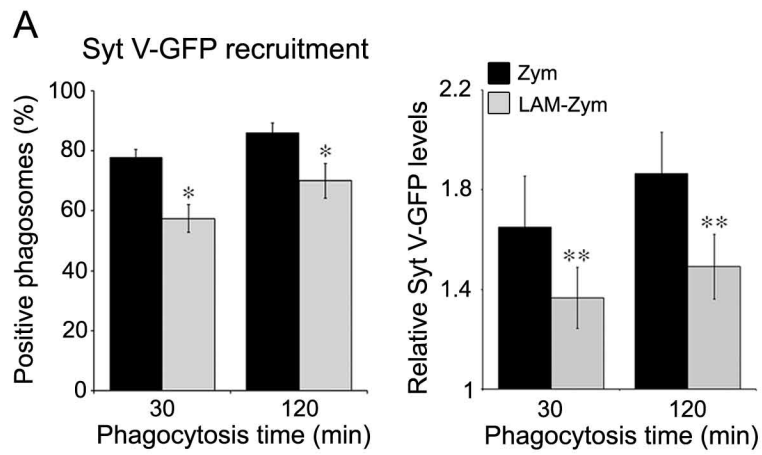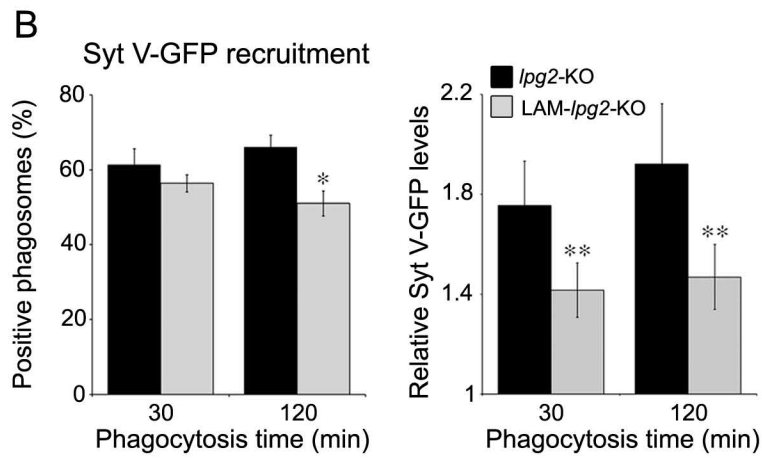

Supplement: Figure S4 — Recruitment of Syt V is prevented on phagosomes containing particles coated with the Mycobacterium tuberculosis lipoarabinomannan. A and B, SytV-GFP cells were allowed to internalized Zym or LAM-Zym (A), lpg2-KO or LAM-lpg2-KO (B) for 30 min or 2 h. The presence (A and B, left graph) and relative levels (A and B, right graph) of Syt V-GFP were determined. Three independent experiments were performed and the bars show the standard deviations of one representative triplicate (*, p≤0.05; **, p≤0.005). (0.66 MB PDF) [file ppat.1000628.s004.pdf]
